# Evaluating Procedure Videos to Support Clinical Nurses With Rare Procedures: Impact on Anxiety and Clinical Reasoning in a Pre‐Post Study

**DOI:** 10.1111/jan.70234

**Published:** 2025-09-22

**Authors:** Jacqueline Colgan, Sarah Kourouche, Geoffrey Tofler, Kim Miles, Thomas Buckley

**Affiliations:** ^1^ Faculty of Medicine and Health The University of Sydney Camperdown New South Wales Australia; ^2^ Cardiology, Critical Care Central Coast Local Health District Gosford New South Wales Australia; ^3^ School of Nursing, College of Health and Medicine University of Tasmania Hobart Tasmania Australia; ^4^ Royal North Shore Hospital St Leonards New South Wales Australia; ^5^ Learning & Development Central Coast Local Health District Gosford New South Wales Australia

**Keywords:** acute, cardiology, clinical procedure, clinical support, nurse, nurse education, nursing, patient safety, video

## Abstract

**Aims:**

To evaluate clinical nurses' perceptions and acceptability of procedure videos developed to support them during rare clinical procedures. In addition, a secondary aim was to investigate whether these videos reduce anxiety and enhance clinical reasoning.

**Design:**

A descriptive multi‐methods study within a pre‐post‐implementation evaluation design.

**Methods:**

Seven locally developed procedure videos (non‐invasive ventilation (*n* = 2), temporary cardiac pacing (*n* = 3) and pericardiocentesis (*n* = 2)) were evaluated using questionnaires before and after a 6‐month implementation period at a local health district in Australia. Participants self‐rated their clinical reasoning skills in response to video procedures, mapped against the Clinical Reasoning cycle stages. The Spielberger State–Trait Anxiety Inventory (STAI) assessed anxiety towards rare procedures. Data from open‐ended questions were analysed using inductive content analysis.

**Results:**

A total of 247 participants completed the pre‐implementation questionnaire, and 133 completed the post‐implementation questionnaire. Before implementation, many reported feeling heightened anxiety when faced with rare or infrequent procedures, reporting levels that exceeded commonly accepted clinical thresholds for concern. Content analysis revealed persistent concerns among participants, including the need for support during new procedures and a fear of making errors. Before implementation, participants reported searching online for videos to support undertaking new procedures. Following implementation, most participants reported that viewing the videos enhanced their learning experience and improved their clinical reasoning. Perceived anxiety towards undertaking rare procedures was not significantly different from post‐implementation.

**Conclusion:**

This study highlights the need for timely support during infrequent clinical procedures, as participants reported anxiety about them. It also demonstrates that procedure videos are valued tools for nurses before rare clinical procedures.

**Implications for the Profession and/or Patient Care:**

Rare procedures are linked to nurse anxiety and mixed emotions, some of which may be eustress. Organisations can enhance clinical resources for nursing staff by providing online videos tailored to local practices and context, which many nurses find helpful for improving clinical reasoning when undertaking rare procedures.

**Impact:**

This study highlights the anxiety nurses experience before rare procedures and the significance of incorporating multimedia resources, especially online videos, in nursing professional development for rarely performed procedures. Additionally, it informs employers about nursing staff preferences.

**Reporting Method:**

SQUIRE 2.0 reporting was adhered to.

**Patient or Public Contribution:**

None.

## 1 Introduction

In high‐acuity healthcare settings, nurses may encounter rare but life‐threatening situations requiring immediate action. In medicine, these are sometimes referred to as High Acuity, Low Occurrence (HALO) events rare procedures or workflows. These are infrequent yet demand an expert‐level response, as they involve sight, limb or life‐threatening situations. There is emerging research addressing HALO procedural skill decay in medical officers (Maltby et al. 2023; Reid and Clancy 2013), but this is also pertinent for nurses who may seldom encounter high‐acuity procedures. Without regular exposure, both confidence and competence can decline, whilst anxiety may rise due to the critical nature of the procedure (Walker et al. 2021).

Health organisations support clinical practice by developing policy and procedure guidelines ‘bureaucratic templates’ (Bail et al. 2009), not always helpful when nurses need to instantly process information for emergent, infrequent, new or rare procedures, such as pericardiocentesis (Ramachandran et al. 2022) or temporary cardiac pacing (Ng et al. 2018). When time is of the essence, many nurses will seek resources to help them manage urgent or rare procedures, often via Google, rather than navigating information databases (Fossum et al. 2022).

Video technology is increasingly used to enhance clinical skills and support workplace procedures among registered health professionals (Colgan et al. 2023), though it is still less common than in undergraduate health professional classrooms, where it has been studied for over 30 years (Eaton 1990; Hew and Lo 2018; Ward 1992; Whitten et al. 1998). Additionally, nurses' use of certain technologies has not always been permitted in the workplace (Donelle et al. 2023).

In this study, we informally consulted the Coronary Care Unit (CCU) nursing staff in one regional hospital to determine what support resources they found helpful. Staff requested videos after a nurse noticed medical officers watching an online video before inserting an indwelling urinary catheter (Yuminaga et al. 2017). The staff reported difficulty finding suitable videos online for cardiac procedures. This was unsurprising as the quality of healthcare information available on YouTube varies widely (Camm et al. 2018; Duman 2020; Kılınc et al. 2018; Madathil et al. 2015), may not be evidence‐based or relevant to local procedures and circumstances (Aras et al. 2023; Demi̇rbağ and Baysal 2023; Mayer et al. 2023; Savran et al. 2023).

The benefits of using videos to support registered nurses with rare procedures in the workplace is an emerging field of research (Colgan et al. 2023; Yeakel et al. 2025). This research expands the body of knowledge, but there are limited studies on this subject. Evidence of improved skills and knowledge from video usage would benefit both nurses and patients in all healthcare settings. Procedure videos for low‐volume, high‐risk procedures have been used in initiatives such as the Low‐Dose, High‐Frequency (LDHF) strategy in nursing education in developing countries (Amee et al. 2020). LDHF interventions have been used successfully for neonatal resuscitation for medical students remotely (Mediratta et al. 2025) and via video for midwives (Umoren et al. 2025).

The study highlights the affective reactions of nurses to unfamiliar procedures, with recommendations for improving procedural support. Our study aimed to determine the acceptability of locally developed videos to support nurses before rare clinical procedures. In addition, a secondary aim was to investigate whether these videos reduce anxiety and enhance clinical reasoning.

## 2 Methods

### 2.1 Study Design

The study employed a pre‐post design. The research was guided by implementation science and behaviour change theory, including the Theoretical Domains Framework (TDF), which has been previously used in this context in both developed (Gawthorne et al. 2025) and developing economies (Zhou et al. 2022), and helps to explore barriers and enablers to behaviour change (Atkins et al. 2017).

### 2.2 Development of the Procedure Videos

To address identified risks in practice, we deliberately focused on rarer, skilled procedures rather than routine or lower‐complexity tasks. This decision reflects the reality that such procedures are sometimes performed outside of regular working hours, when fewer support staff are available and reliance on clinical judgement is heightened. By prioritising these less frequent but critical scenarios, we aimed to support capability building.

Funded by a New South Wales (NSW) Nursing and Midwifery Innovation Scholarship, the Cardiology Care Unit (CCU) nursing team created seven instructional procedure videos over 17 months. As there was no video development guidance available at the time, the team used a structured eLearning resources development process (Health Education and Training Institute 2017). Development aims were that videos should be under 5 min in duration and feature key messages and a visual simulation that integrated the specific procedure's ‘how’ and ‘why’ (Cheung et al. 2018).

The project team identified specific topics to develop videos for emergency cardiology procedures, highlighting the nursing role within the procedure team, such as:

1. Nursing role in assisting with inserting temporary cardiac pacing wires for life‐threatening bradycardia.
2. Nursing role in assisting with performing emergency pericardiocentesis to reduce life‐threatening fluid build‐up around the heart by pericardial drain insertion.
3. Nursing role of setting up urgent non‐invasive ventilation (NIV) for life‐threatening acute cardiogenic pulmonary oedema.

Video development had three main steps:

1. Script development: Key messages were derived from expert opinions and existing procedural guides, transformed into voiceover scripts, and refined through feedback from stakeholders, including Nurse Managers and the Director of Cardiology.
2. Filming: The team organised logistics for filming, creating shot and equipment lists to ensure the correct items were available and used. Hospital volunteers were recruited for patient roles, and filming took place over 3 days, with subsequent editing by a digital learning consultant.
3. The finalised videos underwent a review by subject matter experts and stakeholders to ensure compliance with clinical standards. Completed in late 2019, the videos included closed captions in line with institutional standards.
4. The final video catalogue, detailing runtime, key messages and Uniform Resource Locator (URL), is included in the Files [Link], [Link], [Link] (File S1)

The development of the implementation strategy will be reported separately, outlining the collaborative efforts of the clinical and academic video development team along with key leadership stakeholders. Steps included identifying barriers and facilitators through analysis of the pre‐implementation data. The implementation strategy involved education, role modelling, clinical champions, and training. After iterative feedback and refinement, senior management and infection control leaders approved the final plan, and implementation began in October 2020.

The videos were promoted through educational sessions and branded mousepads. The study videos reflected procedures only performed in cardiology, emergency and intensive care departments. However, they were made available via the hospital intranet and learning management system, which all staff can access.

### 2.3 Sample and Setting

The study was conducted in an outer metropolitan local health district (LHD) in NSW, Australia. Acute care services are provided in two teaching hospitals which are located approximately 35 km apart. The two hospitals have 588 and 323 beds, with the larger hospital offering interventional cardiology services (Central Coast Local Health District 2023). At the time of the study, approximately 500 nurses worked across the target areas of cardiology, emergency, and intensive care departments where the procedures could be performed.

### 2.4 Recruitment

The data were collected using pre‐ and post‐implementation questionnaires using the REDcap web application (Harris et al. 2019). Data were collected before (January–March 2020) and after the implementation period (March–October 2021). The acute care directorate's nursing unit managers emailed nursing staff, with reminder emails sent 1 month after the initial invitation. Before implementation, the project was extensively promoted through in‐person education sessions across all critical care units. However, due to COVID‐19 restrictions, in‐person promotion of the post‐implementation questionnaire was prohibited and could only be conducted via email. All questionnaires were completed anonymously, and participation was considered consent.

### 2.5 Instrument Development and Design

Both the pre‐ and post‐implementation questionnaires were developed based on the 14 domains of the Theoretical Domains Framework (TDF) (Atkins et al. 2017), with questions designed and mapped according to these TDF behaviour‐related domains (Cane et al. 2012). The TDF consists of 14 validated domains, on which the instrument was based: Knowledge, Skills, Social/Professional Role and Identity, Beliefs about Capabilities, Optimism, Beliefs about Consequences, Reinforcement, Intentions, Goals, Memory and Decision Processes, Environmental Context and Resources, Social Influences, Emotion and Behavioural Regulation (Cane et al. 2012). The questionnaires consisted of both quantitative and open‐ended qualitative questions.

The pre‐ and post‐implementation instruments (pre‐27, post‐22‐item tools) were adapted from a previously published study (Kourouche et al. 2019) and based on the 14 domains of the TDF. Permission was obtained to adopt the questionnaire, with minor adaptations made to ensure it was relevant to our study population. The original author of the questionnaire was part of the research team, contributing to its refinement and application in this study. While the original question stems were retained, the individual questions were adapted to focus on video‐related procedures (File S2).

The post‐implementation questionnaire was created based on the pre‐questionnaire, but after initial demographics were revised to gather feedback from participants who had viewed the videos. Questions about the video were framed on the clinical reasoning cycle stages (Levett‐Jones et al. 2017; File S1). Both pre‐ and post‐questionnaires included sections which are described further below on (i) participant characteristics, (ii) participants' perceptions of clinical procedure resources (written and video), (iii) perceived state anxiety, and (iv) clinical reasoning (post only).

#### 2.5.1 Participant Characteristics

The initial sections captured participant demographic characteristics, including gender, age, and years of clinical experience.

#### 2.5.2 Participants' Self‐Reported Use of Procedure Guidelines

Nurses' perceptions regarding written and video procedures, as well as their acceptability and impact on time and patient care, were similarly rated on a 5‐point Likert scale. Participants were asked to report their self‐reported use of clinical procedure guidelines at work (daily, weekly, monthly, yearly and never). Participants were asked to report if they had ever searched online for information about clinical procedures for education or clinical support and, if so, to list the sources they utilised. The effectiveness of video procedures was evaluated using a 5‐point Likert scale, ranging from 1 (strongly disagree) to 5 (strongly agree) points. Additionally, participants were asked to identify the most common reasons for using clinical procedures.

#### 2.5.3 Perceived State Anxiety

Perceived anxiety related to undertaking rare procedures was measured by using Spielberger's state anxiety inventory (Spielberger 1995). This scale's reliability and validity have been established to measure perceived anxiety among registered nurses (Brandford and Reed 2016) and nursing students (Turner and McCarthy 2017). Participants were asked to rank their anxiety on a 4‐point Likert scale (range: not at all/1 to very much so/4 points) on 20 items expressed as statements related to how they feel before a new procedure. Responses are scaled values, with a score of 4 reflecting elevated perceived anxiety levels. For 10 specific statements, higher scores indicate reduced anxiety, and the scores are inverted. The State–Trait Anxiety Inventory (STAI) scale ranges from 20 to 80 points, with scores of 20 points indicating no anxiety and scores above 40 points indicating clinically significant anxiety levels (Spielberger 1995).

#### 2.5.4 Clinical Reasoning

The impact of using videos on clinical reasoning was measured on a 5‐point Likert scale (range: disagree strongly/1 point to agree strongly, good/5 points). The questions had a consistent stem ‘Viewing a video assisted me to’ followed by one of the clinical reasoning stages, i.e., consider the patient situation, collect cues/information, process information, identify problems/issues, establish goals, take action, evaluate outcomes and reflect on the process and new learning (Levett‐Jones et al. 2017). Higher scores reflect stronger agreement with each item, indicating greater perceived cognitive support from the video in relation to specific stages of the Clinical Reasoning Cycle.

### 2.6 Data Collection

Study data were collected and managed using REDcap, hosted by the University of Sydney. REDCap is a secure, web‐based software platform that supports data capture for research studies (Harris et al. 2019). Participants were prompted to answer all questions to progress and minimise missing data. Consent was obtained initially, as outlined in the introduction and research information sheet. A reminder email was sent through initial networks after 2 weeks.

### 2.7 Data Analysis

Quantitative data were analysed using SPSS IBM V 29. Participant characteristics were presented, for example, mean standard deviation (SD) for continuous variables age, or frequencies and percentages (*n* (%)) for categorical variables such as gender, site and role. Responses were presented with a mean (SD) or median interquartile range (IQR), depending on the appropriate data distribution or proportion of data. Anxiety scores were compared from pre‐ to post‐implementation using independent samples Student *t*‐tests. As the responses were collected anonymously, the data were treated as independent samples. Normality testing was performed, and the data were found to be approximately normally distributed, thus satisfying the assumptions required for the *t*‐test. In this study, the STAI's state anxiety subscale demonstrated good internal consistency, with Cronbach's alpha values of 0.91 at pre‐implementation and 0.70 at post‐implementation. The comparison utilised descriptive statistics and a chi‐square test in addition to the *t*‐tests. Correlation analysis was conducted using Pearson's correlation coefficient (*r*) to assess the strength and direction of linear relationships between individual item scores and variables of age and gender. For continuous variables, statistical significance is indicated by a *p* value < 0.05 (Eldredge et al. 2016).

The qualitative data collected from open‐ended questions were analysed using inductive content analysis (Graneheim and Lundman 2004). The questionnaire responses were organised according to their respective sections. Each narrative was thoroughly examined multiple times to understand its overall significance, and emerging categories were noted throughout this review process. Subsequently, the data were analysed using NVivo software (Release 1.7.1; QSR International 2020) and allocated according to categories and subcategories with similar meanings. The first author conducted these steps with co‐authors (SK and TB) to establish the trustworthiness of the qualitative data analysis. Once the content categories were finalised, they were mapped in a diagram and cross‐checked. This was monitored through a reflexive approach involving academic supervision and critical discussions with the research team to ensure saturation progress, reduce bias, and enhance data richness.

### 2.8 Ethics

The study received research governance approval through the Governance Committee Exempt Low Negligible Risk Research application (LNR: 0120‐007C) on 22 April 2020. Participation in the study was voluntary, and participants could skip questions or exit at any time. All responses were anonymous, and no incentives were offered. Anonymous questionnaires ensured data trustworthiness and minimised bias, as the research team was local staff. This approach allowed participants to give honest feedback without concern about professional relationships or project ties. It took place in a Central Coast Local Health District, NSW, Australia, which provides public health services to 358,826 people (New South Wales Ministry of Health 2024). The research team analysed pre‐implementation data, submitted an amendment to the LNR for the post‐implementation phase of the study, and received approval on 3 September 2020. This study used the SQUIRE 2.0 reporting guideline (Ogrinc et al. 2015) (File S1).

### 2.9 Context of Procedure Video Implementation

The final approved videos were ready in late 2019. During the pre‐implementation phase (March–May 2020), NSW faced the initial wave of the COVID‐19 pandemic, prompting rapid public health interventions, lockdowns, and urgent reconfiguration of healthcare delivery (Basseal et al. 2023). This period saw pressure to expedite the release of remote education materials, including procedure videos, to support workforce redeployment, particularly in the area of respiratory support for COVID‐19 patients (Wynne et al. 2021).

The official go‐live on 15 October 2020 coincided with a period of relative pandemic suppression, allowing for broader implementation between October 2020 and March 2021 under COVID‐19 safety protocols, through a combination of face‐to‐face promotion and digital engagement strategies. The post‐implementation period (April–September 2021) coincided with the surge in the Delta variant in NSW, which caused considerable strain on healthcare systems and staff (Hutchinson et al. 2023). Minor pandemic‐driven constraints and adaptations occurred across all study phases, shaping the timing of the study and the intervention, as well as the questionnaires and the strategic value of the interventions within rapidly evolving local and state clinical priorities.

## 3 Results

### 3.1 Participant Characteristics

The analysis included 248 pre‐ and 133 post‐valid responses, with response rates of 49.8% and 26.6% respectively. The sample characteristics are presented in Table 1.

**TABLE 1:** Participant characteristics at pre‐ and post‐implementation.

Participant characteristics	Pre (248)	Post (133)
Age in years mean (SD)	40.9 (10.7)	40.75 (10.1)
Gender	***N* (%)**	***N* (%)**
Female	208 (83.9)	113 (85.6)
Male	39 (15.7)	19 (14.4)
Did not state gender	1 (0.4)	0
Site		
Hospital A	167 (67.6)	86 (64.7)
Hospital B	75 (30.4)	45 (33.8)
Did not state	5 (2.0)	2 (1.5)
Role		
Registered nurse	159 (64.1)	90 (67.2)
Clinical nurse specialist	25 (10.1)	14 (10.6)
Clinical nurse educator	15 (6.0)	13 (9.8)
Nurse unit manager	10 (4.0)	1 (0.8)
Enrolled nurse	9 (3.6)	5 (3.8)
Clinical nurse consultant	9 (3.6)	5 (3.8)
Nurse manager	6 (2.4)	2 (1.6)
Nurse practitioner	3 (1.2)	0
Nurse educator	1 (0.4)	0
Student nurse	0	2 (1.6)
Did not state	11 (4.6)	1 (0.8)
Role		
Time in current role years mean (SD)	7.01 (6.4)	7.4 (6.2)
Specialty		
Cardiology	119 (48.4)	65 (48.9)
Emergency	58 (22.8)	36 (27.1)
Intensive care	37 (15)	17 (12.8)
Other	34 (13.8)	15 (11.3)
Renal	8 (3.2)	0
Medicine	6 (2.4)	4 (3.2)
Neurology	6 (2.4)	0
Med/surgical ward	3 (1.2)	0
Casual nursing pool	3 (1.2)	1 (0.7)
Respiratory	2 (0.8)	8 (6)
Community	2 (0.8)	1 (0.7)
Mental health	1 (0.4)	0
Nursing directorate	1 (0.4)	0
Clinical placement	0	1 (0.7)
Did not state	2 (0.8)	0

Abbreviations: *N*, number of responses; SD, standard deviation.

### 3.2 Pre‐Implementation Self‐Reported Use of Procedure Guidelines

Most study participants reported valuing clinical procedure documents, with over 95% (*n* = 232) strongly agreeing/agreeing. Further, over 77% (*n* = 191) reported accessing written clinical procedures at least weekly. Most participants were confident (strongly agreed/agreed) in locating (91.8%, *n* = 201), understanding (93.6%, *n* = 205) and applying (91.4%, *n* = 200) clinical procedure resources in their practice.

**FIGURE 1:**
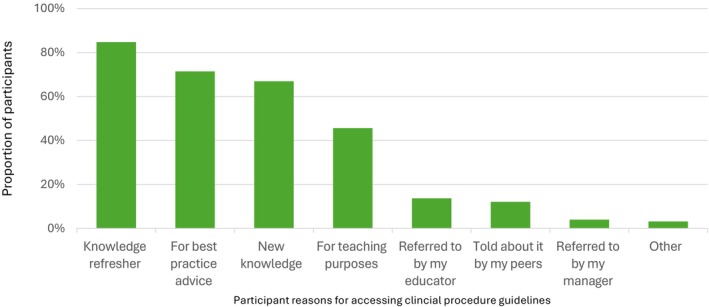
Participant responses to statement: ‘Please select why you are most likely to search the intranet about clinical procedures’. Other included: developing education sessions, searching for specific clinical trial interventions, performing mandatory training, investigating clinical incidents, checking for updates, and searching for information while reviewing or writing new procedure guides.

Participants selected options from a list of seven reasons for accessing written clinical procedures. The most reported reasons were to refresh knowledge, obtain best practice advice, or gain new knowledge (see Figure 1).

### 3.3 Pre‐Implementation Beliefs About Video Procedures

Before the study, videos were implemented, participants rated the impact of procedure videos on their future workload, and 32% (*n* = 67) agreed/strongly agreed that video procedures would not affect workload (48.3%, *n* = 101/210) disagreed strongly/disagreed, and 9.5% (*n* = 20/210) indicated that video procedures would increase their workload. Additionally, 81% (*n* = 171) expressed that they would be more likely to use video procedures if they were available. Many participants believed that the videos would enhance their clinical skills (81.3%, *n* = 170), improve patient outcomes (88.6%, *n* = 186), simplify access to information (86.6%, *n* = 182), and ultimately improve overall healthcare (84.8%, *n* = 178).

### 3.4 Pre‐Implementation Self‐Reported Use of Video

**FIGURE 2:**
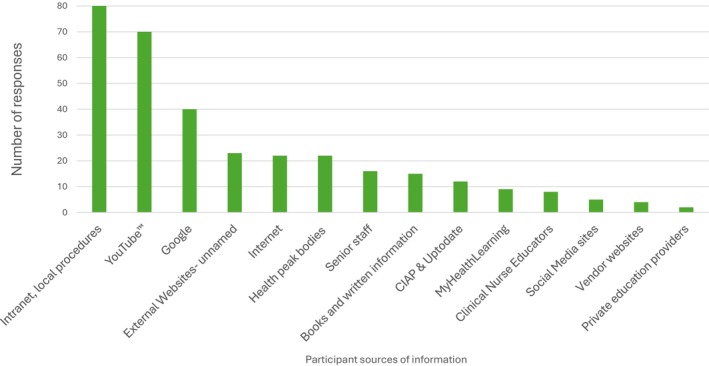
Reported sources of procedure‐related information seeking.

Prior to implementation, 70% of participants (*n* = 143/206) reported having previously searched online for videos containing clinical procedure information (sources listed in Figure 2). Most participants valued having access to these videos, with 88.2% (*n* = 186/211) indicating their importance, and 91.8% (*n* = 192/209) believing that the videos would enhance their understanding of the procedures. Participants also felt that videos would help improve their recall of procedure steps (85.6%, *n* = 178) and clarify their role in the procedures (81.8%, *n* = 171).

### 3.5 Post‐Implementation Participants' Self‐Reported Use of Study Video Procedures

In the post‐implementation questionnaire, 11 (9%) participants exited at the end of Section Two as they had not viewed the study videos. The non‐viewing group was older (mean age 43.7 years vs. 40.4 years) and more experienced (mean time in current role, 10.4 years vs. 7.1 years). Notably, this group still valued video procedures (90.1%, *n* = 11 vs. 96.7%, *n* = 122).

A total of 122 participants viewed the study videos. Among them, 91.8% (*n* = 112) had watched at least one clinical procedure video, averaging 4.5 videos each. The most viewed were pericardiocentesis procedure (70%, *n* = 86) and non‐invasive ventilation (NIV) set‐up (69.6%, *n* = 85). Over 90% of participants agreed/strongly agreed that viewing a video enhanced their learning experience (94%, *n* = 112), improved their memory or recall of procedures (98%, *n* = 117), and made the process of learning quicker and easier (93%, *n* = 112). Regarding time efficiency, 88% (*n* = 102) of participants agreed/strongly agreed that watching a video procedure was timesaving. When asked if the video would prompt them to change their practice, 62% (*n* = 74) agreed/strongly agreed.

### 3.6 Pre‐Post Implementation Perceived State Anxiety When Undertaking a New Procedure

Participants' (*n* = 211 vs. *n* = 110) mean perceived anxiety related to undertaking a new procedure did not change significantly from pre‐ to post‐implementation (42.20 (SD = 9.31) vs. 41.43 (SD = 9.31) points, *p* = 0.43). Both pre‐ and post‐implementation scores were slightly above the accepted cut‐off level of 40 points for significant anxiety (Spielberger 1995).

Younger age (*r* = −0.13, *p* = 0.04 vs. *r* = −0.134, *p* = 0.02) and a lower number of years spent in their current role (*r* = 0.53, *p* < 0.001 vs. *r* = 0.53, *p* < 0.001) were significantly correlated with higher levels of perceived anxiety both pre and post implementation (*r* = 0.53, *p* < 0.001). Further, female participants reported significantly higher levels of anxiety related to undertaking new procedures compared to male participants (mean score 43.05 [SD 7.62] vs. 37.2 [SD 8.83] points, *p* = 0.008 vs. 43 [SD = 7.61] vs. 37.20 [SD = 8.82] points) pre and post implementation.

### 3.7 Content Analysis of Open‐Ended Responses to Perceived State Anxiety When Undertaking a New Procedure

**FIGURE 3:**
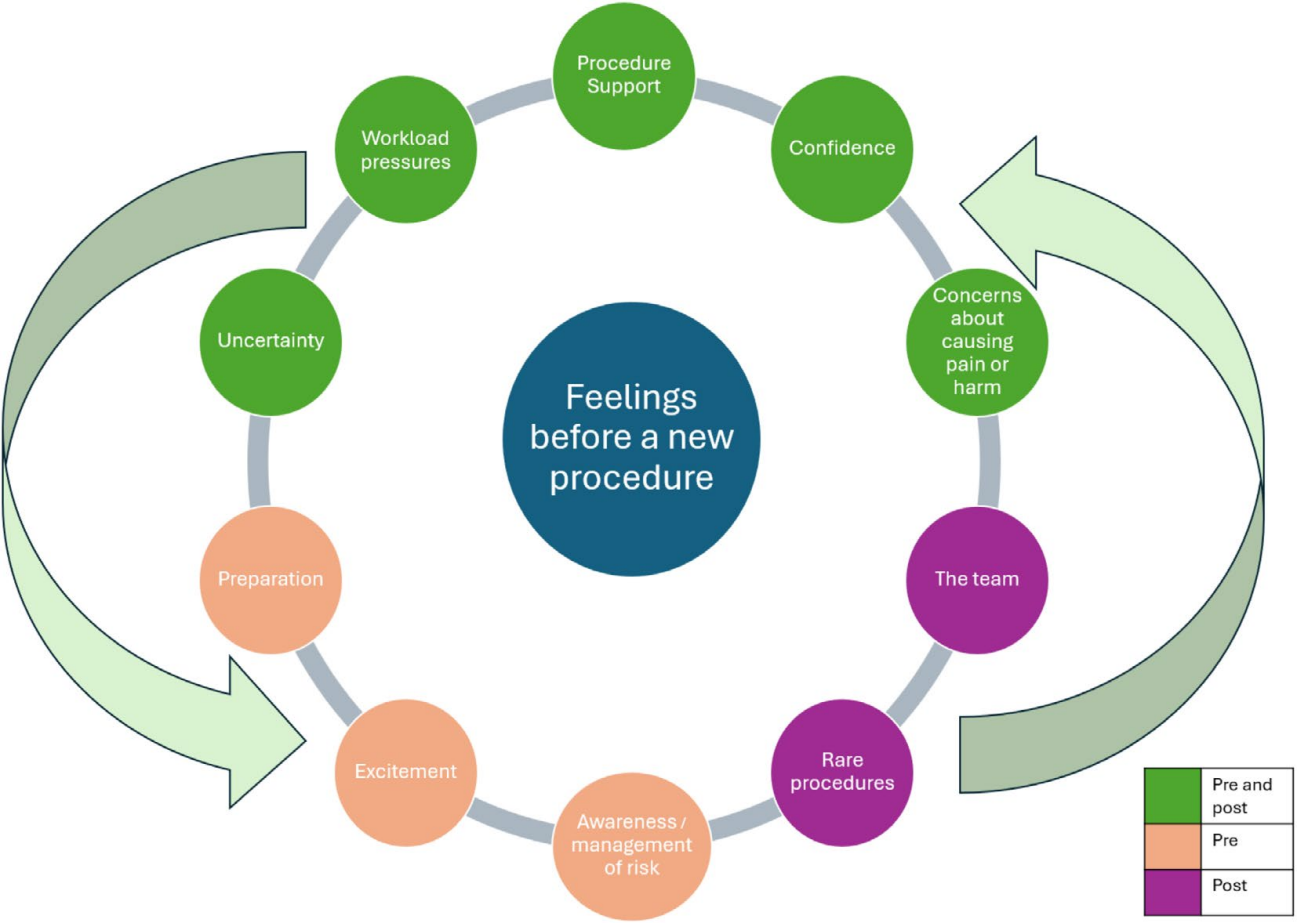
Pre‐post inductive content analysis categories of participant feelings when performing new clinical procedures.

Analysis of open‐ended responses (pre; *n* = 73, 29.4% vs. post; *n* = 51, 41.8%) revealed participants' opinions on how they felt prior to undergoing the new clinical procedure procedures. After an inductive content analysis was performed for the responses received, ten major categories were identified regarding how participants felt about performing new procedures. Five common categories emerged across both time points, indicating consistent areas of concern or interest (Figure 3).

#### 3.7.1 Procedure Support

Comments supported the quantitative findings that support for procedures was important, even for experienced staff such as Clinical Nurse Specialists (In Australia, a Clinical Nurse Specialist is a registered nurse who has advanced expertise in a specific area of clinical practice), if it was a new procedure, as exemplified by the quote below:

A new procedure always gives anxiety and fear if you are not well supported by senior staff or educators. But I am confident to learn new procedures and improve my skills. It always needs training and to be clear about policies before doing it. I always prefer to watch educational videos/procedure videos before doing any procedures.

#### 3.7.2 Confidence

Participants frequently emphasised the confidence that comes with experience when performing procedures, particularly for the first time:

After 29 years of nursing, I am confident in performing procedures, even if new. I am aware of the support around me in policies and procedures, video links and other staff.

#### 3.7.3 Concerns About Causing Pain or Harm

A recurrent thread across the data was concern for patients and preventing harm, as one participant described:

When attempting new procedure, there is a level of fear of doing it wrong and how that may impact patients; occasionally, I feel excited to learn something new, but that is usually only if it is an extension of a skill that I am already comfortable with.

#### 3.7.4 Uncertainty

Responses conveyed a blend of emotions, positive sentiment towards learning opportunities, but as described previously, concern about the potential for error:

Mixed emotions. Appreciative learning something new and being able to learn and grow and hopefully educate other colleagues. At the same time nervous, as you have little knowledge in the skill and could make an error without realising it. Hence why it's important to have a senior watch and challenge you with questions without judgement.

#### 3.7.5 Workload Pressures

Extrinsic factors such as time constraints and staffing levels also affected participants' experience of procedural support:

If able to access proper clinical procedure guidelines, then I don't feel anxious. But when I am not able to look through the guidelines (to refresh my knowledge) due to a lack of time/not enough staffing, etc., I feel nervous.

In addition to these, three unique categories were identified in the pre‐implementation responses:

#### 3.7.6 Awareness/Management of Risk

I always think of the worst outcome and try to prevent this from happening. I worry I will complete the procedure incorrectly and result in harm to my patient. Being in an unknown situation/attempting a new procedure makes me feel very anxious and uncomfortable; I don't like to feel rushed to ensure I do the task correctly.

#### 3.7.7 Excitement

While many participants voiced mixed emotions, a smaller group conveyed pure enthusiasm for engaging with new learning experiences:

I love learning new procedures and feel pleasant if something new.

#### 3.7.8 Preparation

Responses in this category highlighted proactive planning by reviewing support materials, which included watching a video:

A new procedure always gives anxiety and fear if you are not well supported by senior staff or educators. But I am confident to learn new procedures and improve my skills. It always needs training and to be clear about policies before doing it. I always prefer to watch educational videos/procedure videos before doing any procedures.

Finally, two additional categories emerged in the post‐implementation responses, which may reflect new insights or experiences that participants gained from viewing the video(s).

#### 3.7.9 Rare Procedures

Concerns were expressed about the lack of regular exposure to procedures, which was a factor, as one participant explained:

Anxious about procedures that are not everyday business.

One Clinical Nurse Specialist also highlighted the internal pressure experienced:

Being out of practice, knowing the theory, but I haven't had enough clinical practice.

Another highlighted how pressure is also extrinsic and relates to the critical timing for the procedure:

Some new procedures are done under duress and with urgency.

One participant explained that rareness and lack of exposure to procedures related back to concern for patients:

I have confidence in my own knowledge, or lack of, and in seeking to rectify the lack. I am also confident in my abilities and high level of nursing skill, so the more negative feelings related to having not done the procedure before or in a long time and worrying that I do not do the best for the patient.

#### 3.7.10 The Team

A small number of participants reflected specifically on the role of the team for procedure support:

The team in which you perform a new procedure also affects how you feel—i.e., a supportive team reduces anxiety and stress.

The importance of Clinical Nurse Educators as members of the team for procedure support was also acknowledged:

Depending on the situation, and if it is a new procedure, I like to have educators available.

### 3.8 Post‐Implementation Clinical Reasoning

**FIGURE 4:**
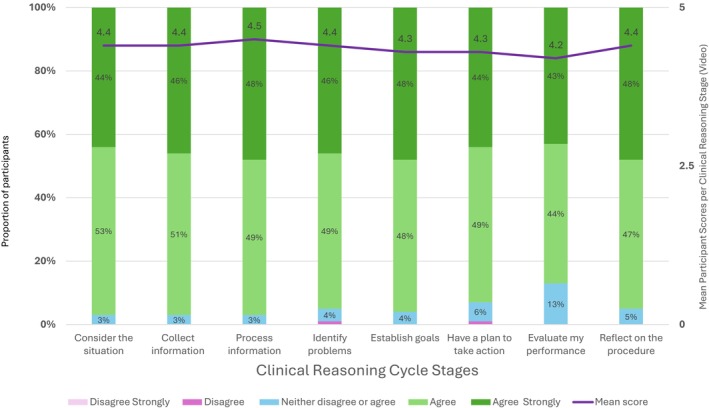
Participant responses to question stem: ‘Using a clinical video procedure assisted me to…’.

Ninety percent (90%) of respondents strongly agreed that watching a video assisted them in aspects of patient care, such as gathering and analysing information, identifying issues and setting goals. Additionally, most participants reported that the videos helped them evaluate and reflect on procedures (Figure 4).

### 3.9 Participant Suggestions for Future Procedure Videos

Nineteen suggestions were received for future potential video topics, comprising three main categories (Table 2).

**TABLE 2:** Category generation for survey question: ‘Do you have other specific suggestions for procedural videos you would find useful?’

Categories	Quotations from participants (edited for typographical errors)
Airway and breathing	Chest tubes—insertion and taping High‐flow nasal prong therapy/high‐flow paediatrics Intubation/surgical airway Intercostal catheter (ICC) insertion and management of underwater seal drain (UWSD) Tracheostomy Tracheostomy management UWSD management
Circulation	Arterial line set‐up/troubleshooting Intraosseous (IO) insertion and securing of umbilical catheters Peripherally inserted central catheter (PICC) dressing and line change Portacath access Preparing for cardioversion Would like more on COACHED (advanced life support cognitive aid)—continue compressions, oxygen away, all others away, charging, hands off, evaluate, defibrillate or disarm; art line set‐up insertion and management
Other—clinical support	Ascites tap Some real‐life photos/examples of possible problems, such as trauma/infection at the drain site Vacuum assisted closure (VAC) dressings

## 4 Discussion

The study reveals that rare procedures induce anxiety and mixed emotions in nurses due to factors such as unfamiliarity and the patient's critical condition. It highlights a significant and novel finding: little has been documented about nurses' experiences with rare procedures, with previous research predominantly focused on medical officers (Cassidy et al. 2020; Celentano et al. 2019; Maltby et al. 2023; Srinivasa et al. 2020). This study demonstrates the importance of addressing nurses' unique perspectives and emotional challenges associated with rare procedures. This anxiety is often linked to the fear of making mistakes. Nurses are concerned about poor procedural outcomes, which could be considered secondary traumatic experiences, as noted by Watson et al. (2025).

The mixed feelings and anxiety reported by participants should not be viewed as unexpected. This may reflect a form of eustress, a positively appraised stress response that can enhance short‐term cognitive and physiological readiness for the procedure (Mithen et al. 2023). Eustress arises when individuals interpret a potential stressor as within their coping capacity, leading to heightened engagement rather than impairment (Le Fevre et al. 2003). Moderate stress can boost motivation and may be helpful in the short term, but prolonged pressure without support can lead to burnout (Galanis et al. 2021). Further, the level and refractoriness of anxiety still warrant further investigation in future studies.

It is possible that the intervention videos did not alleviate anxiety, not necessarily due to design limitations, but because the anxiety itself may reflect an adaptive, performance‐related arousal state‐‘the implicit anxiety of caring’ (Allan 2017, 300). Nurses caring for cardiac patients requiring lifesaving procedures may experience elevated anxiety levels comparable to those reported when caring for COVID‐19 patients (Fernandez et al. 2021; Savci et al. 2021). The link between being younger, less experienced, and higher anxiety levels aligns with findings showing that younger nurses, particularly those with less experience, often report elevated anxiety (Hegney et al. 2014). Additionally, during the COVID‐19 pandemic, the stressors of the nursing profession disproportionately affected younger and less experienced nurses (Grasmann et al. 2024; Ulupınar and Erden 2024).

This study's emphasis on rare procedures and nurse anxiety addresses an underexplored area in multimedia resource development. Video is a low‐cost, on‐demand resource and was acceptable to participants. A key finding related to rare procedures is that almost all participants reported that locally developed videos improved their clinical reasoning. An encouraging finding post‐implementation is that nurses have begun to identify the importance of the team for rare procedures. This suggests that our efforts to introduce this have been effective, and we are hopeful that simulation training may serve as a valuable team‐based method to further embed.

This study extends previous research in undergraduate nurse education (Coyne et al. 2018; Forbes et al. 2016; Stone et al. 2020) and demonstrates the benefits of workplace videos for postgraduate nurses. Findings from both quantitative and qualitative data suggest that video was actively sought after, as YouTube was the second most common information source, indicating a possible learning preference and expectation.

Study participants requested more locally developed videos and offered nineteen suggestions for future procedure videos. Suggestions fell into two main categories: airway‐related and circulation‐related procedures, with comments highlighting a lack of regular exposure to these procedures. These suggestions and nurses' information‐seeking patterns may reflect a broader societal shift towards visual communication, signalling the need for change in contemporary workplace clinical educational strategies. Finally, by openly sharing educational videos on publicly accessible platforms, institutions can support remote learning, reduce duplicative efforts, and help mitigate research waste. Such practices democratise knowledge for locations with a need but limited production capacity, fostering sustainability and collaboration in healthcare education.

## 5 Key Findings

- Video procedures are valued.
- Nurses report anxiety about new or rare procedures.
- Video procedures improve clinical reasoning; however, participants' anxiety levels stayed the same, potentially suggesting beneficial eustress that enhances their ability to perform under pressure.

## 6 Recommendations for Future Research

Future research should explore how pre‐procedural anxiety, potentially representing eustress, influences nurses' performance and emotional regulation in rare clinical procedures. Investigations could focus on preparing through simulation‐based preparation. This research supports the existing literature, emphasising the effectiveness of video for developing clinical skills. Further investigation is needed into rare procedures and nurses. The positive feedback from participants and improvements in clinical reasoning underscore the potential of video‐based interventions. Future research should investigate patient perspectives and assess the long‐term effects of video on clinical procedure skills.

The video development process fosters collaboration that enables participants to enhance their skills and develop valuable clinical support resources. Once these resources are in place, future video requests are likely to arise, as observed in our study. Future research should focus on scaling and adapting video procedures in hospitals as a relatively low‐cost intervention for enhancing psychological safety and emotional readiness, which can be made available on demand. Additionally, exploring the integration of simulation and mental rehearsal for rare procedures could be beneficial.

## 7 Implications for Policy and Practice

This research has significant implications for clinical practice, nursing education, and the well‐being of the healthcare workforce.

### 7.1 Clinical Practice

Procedural videos significantly enhance learning and clinical reasoning. This study advocates for incorporating multimedia resources beyond academic settings and routine skills, highlighting their positive impact on clinical reasoning in rare procedures. Videos are cost‐effective and, when published on Health Facility webpages and corporate YouTube channels, promote quality knowledge sharing globally.

Videos of rare procedures offer a valuable opportunity to promote interdisciplinary collaboration through both development and continuous use. After viewing study videos, participants recognised the importance of ‘the team’ for rare procedures. Resources for rare procedures should incorporate the roles that nurses and the wider healthcare team play in preparation, clinical monitoring, and the aftermath of these procedures.

### 7.2 Nurse Wellbeing and Mental Health

While most participants felt more confident after watching a study video, it did not reduce their perceived anxiety about rare procedures. While cardiac arrest and trauma are well‐known high‐acuity events, rare or complex procedures in inpatient units can also cause considerable staff anxiety, necessitating the need for similar psychological safety measures.

### 7.3 Nurses' Continuing Professional Development

Newly qualified nurses often transition from training environments with extensive audiovisual teaching materials to clinical settings that rely on written guidelines. This shift may disrupt their confidence and increase cognitive load during rare or new procedures. To better support new nurses, it may be helpful to combine the video with regular simulation programmes, such as those used for emergency response to clinical deterioration, designed explicitly for rare scenarios.

This study addresses a critical gap in nursing education by focusing on procedures that are rarely performed in cardiac care. This area remains under‐researched in nursing, despite growing interest in the field of medicine. Our findings show that staff were already seeking procedural videos online, highlighting a natural reliance on video as part of ‘just in time’ learning. By developing high‐quality, clinically accurate video resources and embedding them into practice, we demonstrated not only their acceptability but also their potential to improve clinical reasoning. This study advances the evidence base for video as a workplace‐integrated teaching tool for rare procedures in nursing.

### 7.4 Strengths and Limitations

Previous research on this topic has been primarily focused on classroom teaching or patient education. A strength of this study is that it was led by clinicians and undertaken in a clinical environment with postgraduate nurses. Furthermore, the study highlights nurse anxiety, rare procedures as new aspects, and our use of video translates the nursing literature findings in academic settings to workplaces (Coyne et al. 2018; Forbes et al. 2016). In addition, we used an evidence‐based framework for video implementation (Cane et al. 2012) and evaluation (Levett‐Jones et al. 2010). This research has limitations: It was conducted in a single local health district, and the pre‐implementation response rate is within the typical range of 35% to 50% for nursing research (L'Ecuyer et al. 2023). The post‐implementation response rate was 26.6%, representing a 23.2% decrease, which is similar to other studies conducted during the COVID‐19 pandemic (13%–27%) (Krieger et al. 2023; Kursumovic et al. 2021; Shiyab et al. 2023).

This lower response rate is also proportional to the number of individuals who viewed the videos during the implementation period. As a result, it may limit the generalisability of the findings, as the views of non‐respondents may differ significantly from those of participants. This study faced challenges during the COVID‐19 pandemic. Specifically, in‐person promotion of the post‐implementation questionnaire was not possible due to staff restrictions preventing entry into units such as emergency or intensive care units. The study questionnaire could only be promoted via email reminders and to colleagues. Additionally, since data were collected anonymously, the dependence between pre‐ and post‐implementation participants could not be accounted for in the statistical analyses (File S3).

This study influenced early clinical education responses during the pandemic, prompting the creation of targeted procedure videos that covered non‐invasive ventilation in COVID‐19. Following presentation and peer engagement, the videos were shared with health professionals across NSW and internationally, supporting just‐in‐time procedural guidance and service commissioning. Notwithstanding the limitations, the study suggests that video procedures are valued and useful to nurses.

## 8 Conclusion

Nurses report high levels of anxiety when faced with undertaking new or rare clinical procedures, and a high proportion turn to internet video support. Our findings show that locally prepared videos for rare clinical procedures were highly valued, enhanced learning, improved information retention, and improved confidence. Videos can support healthcare professionals during clinical procedures, improving clinical reasoning and thereby patient care. Integrating video‐based clinical support into nursing practice at the point of care is a promising strategy for addressing fears related to new or rare procedures.

## Author Contributions

J.C., S.K., G.T., K.M., T.B. made substantial contributions to conception and design, or acquisition of data, or analysis and interpretation of data. J.C., S.K., G.T., T.B. involved in drafting the manuscript or revising it critically for important intellectual content. J.C., S.K., G.T., K.M., T.B. given final approval of the version to be published. Each author should have participated sufficiently in the work to take public responsibility for appropriate portions of the content. J.C., S.K., G.T., T.B. agreed to be accountable for all aspects of the work in ensuring that questions related to the accuracy or integrity of any part of the work are appropriately investigated and resolved.

## Disclosure

Statistical analysis: The author team includes a statistician, Thomas Buckley, who affirms that the methods used in the data analyses are suitably applied to the data within the study design and context and that the statistical findings have been implemented and interpreted correctly.

## Conflicts of Interest

The authors declare no conflicts of interest.

## Supporting information


**File S1:** jan70234‐sup‐0001‐FileS1.pdf.

**File S2:** jan70234‐sup‐0002‐FileS2.pdf.

**File S3:** jan70234‐sup‐0003‐FileS3.docx.

## Data Availability

The data that support the findings of this study are available on request from the corresponding author. The data are not publicly available due to privacy or ethical restrictions.
