# Supplementary material for: Evaluating Procedure Videos to Support Clinical Nurses With Rare Procedures: Impact on Anxiety and Clinical Reasoning in a Pre‐Post Study
Source: J Adv Nurs. 2025 Sep 22;82(6):5923–36. doi: 10.1111/jan.70234 (PMC13176733; doi:10.1111/jan.70234)
Supplement: Supplementary file 1 — File S1: jan70234‐sup‐0001‐FileS1.pdf. [file JAN-82-5923-s002.pdf]

Pre-Implementation survey

Clinical Procedures Survey

Thank you for taking the time to complete this survey. This survey is the first part of a multi-phased research project to implement and evaluate clinical procedure videos.The purpose of this research project is to obtain information about the use of clinical procedures by nurses.This survey is being conducted by Jackie Colgan Cardiac CNC as part of a research degree at The University of Sydney and Kim Miles Education Consultant Central Coast Local Health District.

This project is being supervised by Sydney University Associate Professor Tom Buckley and Ms Sarah Kourouche.You are invited to participate in this research project because you are a staff member in Central Coast Local Health District.This study has been reviewed and approved by the Central Coast Local Health District Research Governance Office.

Thank you!

If you are completing this survey on a mobile phone we suggest using landscape mode for optimal viewing.

PLEASE NOTE YOU CAN CONTROL THE FONT SIZE FROM ZOOM BUTTONS IN TOP RIGHT-HAND CORNER OF YOUR SCREEN

You will have unlimited time to complete this survey, however, if you log out prior to completion, your responses will not be saved.

Participant Information Sheet

Please take time to read.

[Attachment: "Participant Information Sheet_ v3 24042020.pdf"]

By completing this survey, you will be providing implied consent and confirming that you have read the Participant Information Sheet. This Sheet can be read, saved and printed by clicking the link.

ELECTRONIC CONSENT: Please select your choice below.
Clicking on the "agree" button below indicates that:
• you have ready the above information
• you voluntarily agree to participate
If you do proceed you agree to participate in the research study.

○ Agree

Section 1: Demographics

What is your age? (please answer in years)	____________ (in years)
What is your gender?	○ Female<br>○ Male<br>○ Non-binary/ third gender<br>○ Prefer to self-describe<br>○ Prefer not to say
What hospital site do you currently work at:	○ Gosford<br>○ Wyong<br>○ Woy Woy<br>○ Long Jetty
What is your role at CCLHD?	○ Registered Nurse ○ Clinical Nurse Specialist ○ Clinical Nurse Educator<br>○ Clinical Nurse Consultant<br>○ Nurse Unit Manager ○ Other
If you selected other, please specify:	____________
What clinical specialty area do you work the majority of your shifts in?	○ Cardiology<br>○ Emergency<br>○ Intensive Care<br>○ Other
If you selected other, please specify:	____________
How long have you been in your current role, please answer in years to 1 decimal point (e.g. 1.5 years)	____________ (in years)

Section 2: Knowledge and Skills

The following questions relate to hospital clinical procedure documents, for example, to documents that exist on the central coast local health district intranet site.

A 'clinical procedure' is defined as a set of approved steps for a particular act or sequence of acts. Procedures are based on validated evidence, have a consistent application and inform the clinician reader how to achieve the necessary results.

Please rate you agreement with the following statement: I value written clinical procedures.

- ◯ Disagree strongly
- ◯ Disagree
- ◯ Neither agree or disagree
- ◯ Agree
- ◯ Agree strongly

I access LHD intranet clinical procedures approximately (select one):

- ◯ once per shift
- ◯ once per week
- ◯ once per month
- ◯ once per year
- ◯ never (I don't access clinical procedures)

Please select the reasons you are most likely to search the intranet in relation to clinical procedures (you may select more than one):

- ☐ New knowledge
- ☐ Knowledge refresher
- ☐ For teaching purposes
- ☐ For best practice advice
- ☐ Referred to by my manager
- ☐ Referred to by my educator
- ☐ Told about it by my peers
- ☐ Other

If you selected other, please specify:

____

How important are the following in relation to written clinical procedures (select your response on the scale for each intervention)
Clinical procedures (defined as a set of approved steps for a particular act or sequence of acts) should be...

	Unimportant	Of little Importance	Moderately Important	Important	Very Important
Practical and useful	○	○	○	○	○
Just the right length	○	○	○	○	○
Easy to follow and understand	○	○	○	○	○
Easy to locate and access	○	○	○	○	○

Please rate in order of importance the following qualities from the lowest (1) to the highest (4) in relation to current written clinical procedures.

	1 (lowest importance)	2	3	4 (highest importance)
Practical and useful	○	○	○	○
Just the right length	○	○	○	○
Easy to follow and understand	○	○	○	○
Easy to locate and access	○	○	○	○

What do you believe is the right number of pages for a written clinical procedure (defined as a set of approved steps for a particular act or sequence of acts) ?

____
(number of pages)

Please specify any other aspects that are important to you in relation to the current written clinical procedures: ____________________

I am confident in my ability to (select your response on the scale for each intervention):

	Disagree strongly	Disagree	Neither agree or disagree	Agree	Agree strongly
Locate procedures on the intranet	○	○	○	○	○
Understand written clinical procedures	○	○	○	○	○
Apply written clinical procedures in my practice	○	○	○	○	○

Select your response in relation to your experience with clinical procedures:	Never	Rarely	Sometimes	Very often	Always
I find it easy to remember where to access new clinical procedures	O	O	O	O	O
During my work day I think there are too many clinical procedures to remember	O	O	O	O	O
I find it difficult to recall written clinical procedures in my practice	O	O	O	O	O

Section 3: Facilitators and Barriers

How important are the following environmental factors in helping you to remember clinical procedures (defined as a set of approved steps for a particular act or sequence of acts):

	Unimportant	Of little importance	Moderately important	Important	Very important
Having a list of clinical procedures on the intranet/internet	O	O	O	O	O
A printed copy of the procedure kept in the patient notes	O	O	O	O	O
A guide to show how to access clinical procedures	O	O	O	O	O
Access to intranet computers in the clinical area	O	O	O	O	O
A video resource that show the clinical procedural steps on the intranet	O	O	O	O	O
A poster with QR codes to look up clinical procedure videos on mobile devices	O	O	O	O	O

Please specify any other factors that help you to remember clinical procedures:

____

Please rate how important (or not important) the following factors are in improving your use of clinical procedures (defined as a set of approved steps for a particular act or sequence of acts):

	Unimportant	Of little importance	Moderately important	Important	Very important
Knowing I will be spoken to by a superior if I do not follow recommended clinical procedure steps	○	○	○	○	○
Knowing my practice may be audited	○	○	○	○	○
Feeling that the clinical procedure will make a difference to the patient	○	○	○	○	○
Other staff reminding me too	○	○	○	○	○
Identifying myself as a "change agent"	○	○	○	○	○
Being aware of risks associated with not following a clinical procedure	○	○	○	○	○

Other (please comment) ______________________________

How important are the following educational supports in using/doing a new clinical procedure (select your response on the scale for each intervention)?

	Unimportant	Of little importance	Moderately important	Important	Very important
A short course on material	○	○	○	○	○
Online learning program	○	○	○	○	○
Help on the floor from senior	○	○	○	○	○
In-service on procedure content	○	○	○	○	○
My colleagues reminding me	○	○	○	○	○
An endorsed locally tailored video procedure	○	○	○	○	○
Other - please comment below	○	○	○	○	○

Please tell us any other educational supports you use when using a new clinical procedure (not listed in the previous question): ______________________________

Almost finished! Thank you for your persistence.

When undertaking a new procedure, usually I feel (select all options that apply):

	Not at all	Somewhat	Moderately so	Very much so
I feel calm	○	○	○	○
I feel secure	○	○	○	○
I feel tense	○	○	○	○
I feel regretful	○	○	○	○
I feel at ease	○	○	○	○
I feel upset	○	○	○	○
I am presently worrying over possible misfortunes	○	○	○	○
I feel rested	○	○	○	○
I feel anxious	○	○	○	○
I feel comfortable	○	○	○	○
I feel self-confident	○	○	○	○
I feel nervous	○	○	○	○
I feel jittery	○	○	○	○
I feel "high strung"	○	○	○	○
I am relaxed	○	○	○	○
I feel content	○	○	○	○
I am worried	○	○	○	○
I feel over-excited and "rattled"	○	○	○	○
I feel joyful	○	○	○	○
I feel pleasant	○	○	○	○

In relation to the previous question, what do you think makes you feel this way?

____

Please add your comments:

Section 4: Video clinical procedures

Video is an effective, accessible, and low-cost method of delivering health information messages to a wide audience.

This study proposes the creation and implementation of locally tailored clinical procedure videos (defined as video simulation of clinical procedures with key messages for procedure safety) as an adjunct to written procedures.

Please rate your agreement with the following statement: I would value clinical procedure videos.	○ Not at all<br>○ Somewhat<br>○ Moderately so<br>○ Very much so
Have you ever searched for videos for procedure information related to your work?	○ Yes ○ No
Please describe below all of the sources you have looked for procedure information	______________________________
Where do you think video procedures should be made available (you can choose all that options that apply)?	☐ Hospital Intranet site<br>☐ NSW My Health Learning<br>☐ Downloadable onto personal mobile devices<br>☐ Other
If you choose other, please tell us any other locations you think video procedures should be:	______________________________

Select your response on the scale for each option,
if video procedures are accessible in your health service, it will

	Disagree strongly	Disagree	Neither agree or disagree	Agree	Agree strongly
Improve patient outcomes	○	○	○	○	○
Make your workload higher	○	○	○	○	○
Make your workload lower	○	○	○	○	○
Have no change to your workload	○	○	○	○	○
Make it easier to access information	○	○	○	○	○
Reduce delays when preparing patients for clinical procedures	○	○	○	○	○
Decrease errors in patient care	○	○	○	○	○
Improve overall patient care	○	○	○	○	○
Improve health care process	○	○	○	○	○
be acceptable by managers to view clinical procedure videos	○	○	○	○	○
Result in staff on computers/phones all the time	○	○	○	○	○
increase complaints by staff, relatives and patients about staff watching clinical procedure videos at work	○	○	○	○	○
Result in patients losing confidence in staff if they see clinicians watching clinical procedure videos at work	○	○	○	○	○

Please add any additional comments:

____

If clinical procedure videos were available in Central Coast Local Health District it would:	Not at all	Somewhat	Moderately so	Very much so
Enhance my understanding of the specific clinical procedure	○	○	○	○
Be more value to me than a written clinical procedure	○	○	○	○
Increase my clinical skills	○	○	○	○
Increase my memory / recall of written clinical procedure steps	○	○	○	○
Increase my understanding of my role in a clinical procedure	○	○	○	○
Increase my likelihood to use it	○	○	○	○
Increase my adherence to Central Coast Local Health District procedures	○	○	○	○
Increase my awareness of the clinical risk associated with procedure	○	○	○	○

What is the maximum time length in minutes you believe a video procedure should be in duration?

______________________ (in minutes)

Please indicate your preference for institutional clinical procedures:

- ○ video procedure
- ○ written procedure
- ○ both video and written procedure
- ○ Other, please comment below

Please add any additional comments below about your preferences for clinical procedure support:

____

Thank you from the project team.

Post Video Procedure Implementation Survey

Thank you for taking the time to complete this survey. This survey is part of a multi-phased research project to implement and evaluate clinical procedure videos.The purpose of this research project is to obtain information about the use of clinical procedures by nurses.This survey is being conducted by Jackie Colgan Cardiac CNC as part of a research degree at The University of Sydney and Kim Miles Education Consultant Central Coast Local HealthDistrict.

This project is being supervised by Sydney University Associate Professor Tom Buckley, Dr Sarah Kourouche and Professor Geoff Tofler. You are invited to participate in this research project because you are a staff member in Central Coast Local Health District.This study has been reviewed and approved by the Central Coast Local Health District Research Governance Office.

If you are completing this survey on a mobile phone we suggest using landscape mode for optimal viewing.

You will have unlimited time to complete this survey, however, if you log out prior to completion, your responses will not be saved.

Please complete the survey below.

Thank you!

PLEASE NOTE YOU CAN CONTROL THE FONT SIZE FROM ZOOM BUTTONS IN TOP RIGHT-HAND CORNER OF YOUR SCREEN

Participant Information Sheet

Please take time to read.

[Attachment: "Invitation to Participate Letter_ v1_survey_2.pdf"]

By completing this survey, you will be providing implied consent and confirming that you have read the Participant Information Sheet. This sheet can be read, saved and printed by clicking the link.

ELECTRONIC CONSENT: Please select your choice below.

Clicking on the "agree" button below indicates that:

- you have read the above information
- you voluntarily agree to participate

If you do proceed you agree to participate in the research study.

○ Agree

Section 1: Demographics

What is your age? (please answer in years)

________________________ (in years)

What is your gender?

○ Female
○ Male
○ Non-binary/ third gender
○ Prefer to self-describe
○ Prefer not to say

What hospital site do you currently work at:

- ○ Gosford
- ○ Wyong
- ○ Woy Woy
- ○ Long Jetty

What is your role at CCLHD?

- ○ Enrolled Nurse ○ Registered Nurse
- ○ Clinical Nurse Specialist
- ○ Clinical Nurse Specialist grade 2
- ○ Clinical Nurse Educator
- ○ Clinical Nurse Consultant
- ○ Nurse Unit Manager ○ Nurse Practitioner
- ○ Other

(If you have more than one role, please choose the role you work in the majority of your shifts.)

If you selected other, please specify:

____

How long have you been in your current role, please answer in years to 1 decimal point (e.g. six months or less =0.5 years)

____
(in years)

What clinical specialty area do you work the majority of your shifts in?

- ○ Cardiology
- ○ Emergency
- ○ Intensive Care
- ○ Respiratory Medicine
- ○ Perioperative
- ○ Surgery
- ○ Other

If you selected other, please specify:

____

Section 2: Knowledge and Skills

The following questions relate to clinical procedures presented in video format (referred to as video clinical procedures):

Please rate your agreement with the following statement: I value video clinical procedures.

- ○ Disagree strongly
- ○ Disagree
- ○ Neither agree or disagree
- ○ Agree
- ○ Agree strongly

Please select the reasons you are most likely to search in relation to clinical procedures (you may select more than one):

- ☐ For patient care
- ☐ New knowledge
- ☐ Knowledge refresher
- ☐ For teaching purposes
- ☐ For best practice advice
- ☐ Referred to by my manager
- ☐ Referred to by my educator
- ☐ Told about it by my peers
- ☐ Other

If you selected other, please specify:

____

CCLHD Clinical Procedure Videos

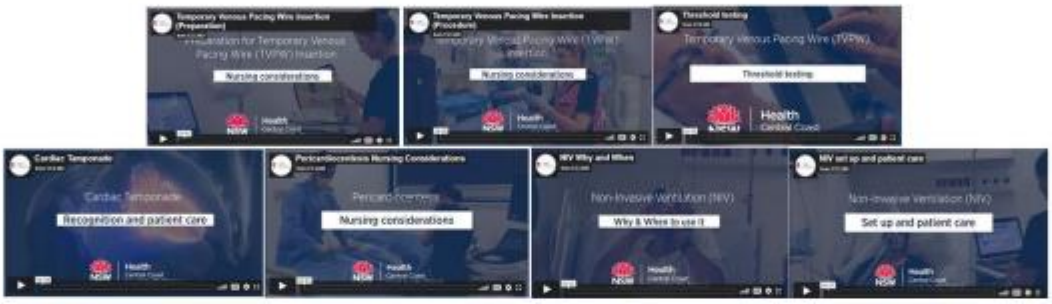

Have you viewed any CCLHD procedure videos?

○ Yes
○ No
(Tip: If you havent viewed all of the videos you can access them at work via the relevant procedure-hyperlink on front page or here: http://intranet.cclhd.health.nsw.gov.au/clinical/AcuteCare/Medicine/cardio/pv/Pages/default.aspx At home or work you can access them via myhealthlearning.)

Please indicate which NIV video you have viewed.

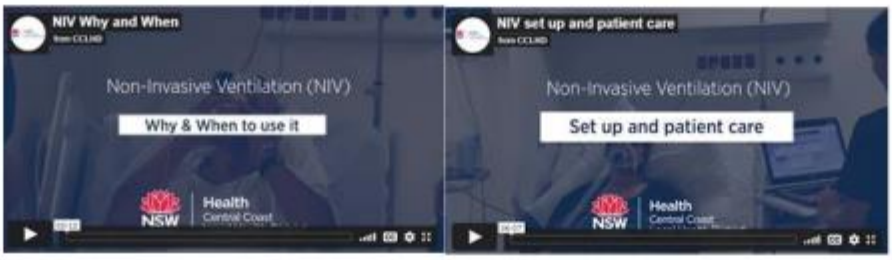

	Yes	No
Video: Why and when to use it	○	○
Video: Set up and patient care	○	○

About the NIV videos

	Disagree strongly	Disagree	Neither agree or disagree	Agree	Agree strongly
Viewing a NIV video procedure assisted me to consider the patient situation	○	○	○	○	○

Viewing a NIV video procedure assisted me to collect visual cues/ information to assist with patient care	○	○	○	○	○
Viewing a video assisted me to process information for NIV procedure	○	○	○	○	○
Viewing a video assisted me to identify problem / issue that could occur when using NIV	○	○	○	○	○
Viewing a video assisted me to establish goals for NIV	○	○	○	○	○
Viewing a video assisted me to have a plan to take action when a problem / issue occurs during NIV	○	○	○	○	○
Viewing a video assisted me to evaluate my performance when using NIV	○	○	○	○	○
Viewing a video assisted me to reflect on the NIV procedure and new learning	○	○	○	○	○

Please add here comments you want to make about about the NIV videos: ______________________

Please indicate which pacing related video you viewed:

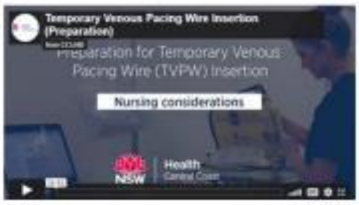

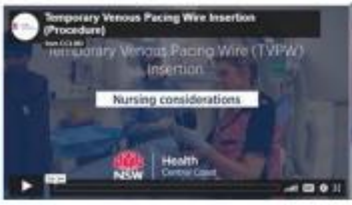

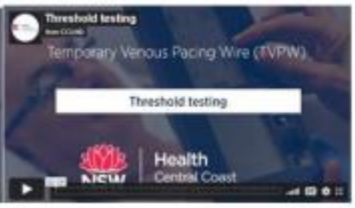

	Yes	No
Video: Temporary venous pacing: preparation	○	○
Video: Temporary venous pacing: procedure	○	○
Video: Temporary venous pacing: threshold testing	○	○

About the pacing videos

	Disagree strongly	Disagree	Neither agree or disagree	Agree	Agree strongly
Viewing a temporary pacing video procedure assisted me to consider the patient situation	○	○	○	○	○
Viewing a video procedure assisted me to collect visual cues/ information to assist with patient care during temporary pacing	○	○	○	○	○
Viewing a video assisted me to process information for temporary pacing procedure	○	○	○	○	○
Viewing a video assisted me to identify problem / issue that could occur during temporary pacing	○	○	○	○	○
Viewing a video assisted me to establish goals for temporary pacing procedures	○	○	○	○	○
Viewing a video assisted me to have a plan to take action when a problem / issue occurs during temporary pacing	○	○	○	○	○
Viewing a video assisted me to evaluate my performance in the temporary pacing procedure	○	○	○	○	○
Viewing a video assisted me to reflect on the temporary pacing procedure and new learning	○	○	○	○	○

Please add here any comments you want to make about the pacing videos: ______________________

Please indicate which video-cardiac tamponade and / or pericardiocentesis procedure you viewed:

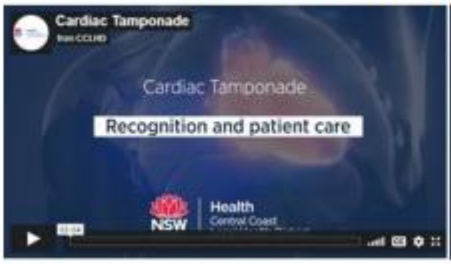

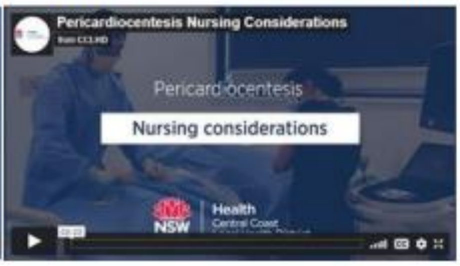

	Yes	No
Video: Cardiac tamponade: recognition and patient care	○	○
Video: Pericardiocentesis: nursing considerations	○	○

About the cardiac tamponade and / or pericardiocentesis video

	Disagree strongly	Disagree	Neither agree or disagree	Agree	Agree strongly
Viewing a video procedure assisted me to consider the patient situation during cardiac tamponade and / or pericardiocentesis	○	○	○	○	○
Viewing a video procedure assisted me to collect visual cues/ information to assist with patient care during cardiac tamponade and / or pericardiocentesis	○	○	○	○	○
Viewing a video assisted me to process information for cardiac tamponade and / or pericardiocentesis procedure	○	○	○	○	○
Viewing a video assisted me to identify problem / issue that could occur in cardiac tamponade and / or pericardiocentesis	○	○	○	○	○
Viewing a video assisted me to establish goals for cardiac tamponade and / or pericardiocentesis procedure	○	○	○	○	○
Viewing a video assisted me to have a plan to take action when a problem / issue occurs during cardiac tamponade and / or pericardiocentesis	○	○	○	○	○
Viewing a video assisted me to evaluate my performance during cardiac tamponade and / or pericardiocentesis procedure	○	○	○	○	○
Viewing a video assisted me to reflect on cardiac tamponade and/ or pericardiocentesis procedure and new learning	○	○	○	○	○

Please add here any comments you wish to make about the cardiac tamponade and / or pericardiocentesis video: ______________________________

Please leave any additional comments about viewing any of the procedure videos here:

____

Using video procedures

I am confident in my ability to (select your response on the scale for each intervention):

	Disagree strongly	Disagree	Neither agree or disagree	Agree	Agree strongly
Locate video clinical procedures on the intranet	○	○	○	○	○
Locate video clinical procedures on myhealthlearning	○	○	○	○	○
Apply video clinical procedures in my practice	○	○	○	○	○
Use of a video procedure increased my confidence that I could manage in the procedure	○	○	○	○	○

Section 3 Barriers and Facilitators to using video procedures

How important are the following factors about video clinical procedures:

	Unimportant	Of little importance	Moderately important	Important	Very important
Realistic simulations with staff who I recognise in locations I am familiar with	○	○	○	○	○
Words on screen to reinforce concepts in the video	○	○	○	○	○
Background music on the clinical procedure videos	○	○	○	○	○
Closed captions on the clinical procedure videos	○	○	○	○	○
Access to intranet computers in the clinical area	○	○	○	○	○
Access to clinical procedure videos on personal mobile devices	○	○	○	○	○

Please add any additional comments about video features below:

____

Section 4: Video clinical procedures

Using a video clinical procedure (Select your response on the scale for each option):

	Disagree strongly	Disagree	Neither agree or disagree	Agree	Agree strongly
Increased my likelihood of reviewing the written document and video about the procedure before performing it	O	O	O	O	O
Increased my adherence to CCLHD procedures	O	O	O	O	O
Reminded me to look up the written procedure	O	O	O	O	O
Increased my memory / recall of clinical procedure steps	O	O	O	O	O
The video made learning about the topic a better experience than I would have had otherwise	O	O	O	O	O
I learned about the topic more quickly and easily because of watching the video	O	O	O	O	O
Video is the best format to present this information	O	O	O	O	O
The video has prompted me to consider changing my practice	O	O	O	O	O
Video procedures should be no longer 5 minutes duration	O	O	O	O	O
Is time-saving for me	O	O	O	O	O
Adds more value to me than what is already clinical available	O	O	O	O	O

Please indicate your preference for institutional clinical procedures:

- O video procedure
- O written procedure
- O both video and written procedure
- O Other, please comment below

Please comment here:

____

Do you have other specific suggestions for procedural videos you would find useful?

____

(Please list ideas or recommendations for videos you would find useful in your clinical practice.)

Almost finished! Thanks for your patience

When undertaking a new procedure usually (select all options that apply):

	Not at all	Somewhat	Moderately so	Very much so
I feel calm	○	○	○	○
I feel secure	○	○	○	○
I feel tense	○	○	○	○
I feel regretful	○	○	○	○
I feel at ease	○	○	○	○
I feel upset	○	○	○	○
I am presently worrying over possible misfortunes	○	○	○	○
I feel rested	○	○	○	○
I feel anxious	○	○	○	○
I feel comfortable	○	○	○	○
I feel self-confident	○	○	○	○
I feel nervous	○	○	○	○
I feel jittery	○	○	○	○
I feel "high strung"	○	○	○	○
I am relaxed	○	○	○	○
I feel content	○	○	○	○
I am worried	○	○	○	○
I feel over-excited and "rattled"	○	○	○	○
I feel joyful	○	○	○	○
I feel pleasant	○	○	○	○

What do you think makes you feel this way? Please add your comments here: ______________________________

Please indicate if you are happy to be contacted in the future to explore themes of video procedures in this survey for group or one on one interview (telephone, skype or in person)

○ Yes
○ No

Please leave your name, contact details and preferred method of contact below:

____
(Name)

____
(Phone number)

____
(Email address)

____
(Preferred method of contact)

Thank you from the project team for completing this survey

9 / 9
