# Supplementary material for: Evaluating Procedure Videos to Support Clinical Nurses With Rare Procedures: Impact on Anxiety and Clinical Reasoning in a Pre‐Post Study
Source: J Adv Nurs. 2025 Sep 22;82(6):5923–36. doi: 10.1111/jan.70234 (PMC13176733; doi:10.1111/jan.70234)
Supplement: Supplementary file 2 — File S2: jan70234‐sup‐0002‐FileS2.pdf. [file JAN-82-5923-s003.pdf]

Topic	Titles & Video length (mm.ss)	Video Key messages	URL
**Non-invasive Ventilation**	1. Why and when (3.13)<br>1. Setup and Patient Care (6.07)	• Goals of NIV therapy<br>• Factors to consider for NIV to be beneficial<br>• What equipment is needed and available<br>• The nurse needs to be familiar with the settings.<br>• How to set the machine<br>• Commencement of NIV and fitting of the mask<br>• Assessing response to NIV<br>• Documentation and observations	https://youtu.be/rTczM0Ik8bw?si=ZthK3smD_Eqq7erM<br>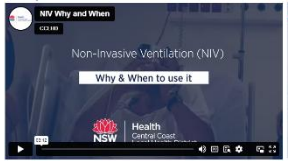<br>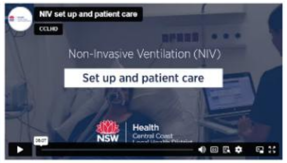
**Cardiac Pacing**	1. Preparation (3.52)<br>2. Procedure (4.34)<br>3. Threshold testing (2.29)	• Preparing for the procedure<br>• Equipment to have ready<br>• Roles - Advanced Life Support Nurse: caring for the patient and clinical monitoring and assist set up pacing box<br>• Roles- Procedural scout nurse: assisting with the procedure requirements<br>• Post procedure management<br>• Patient safety- red flags in pacing	https://youtu.be/A0kjhfzEK24?si=aiTis8apoHEKW-BM<br>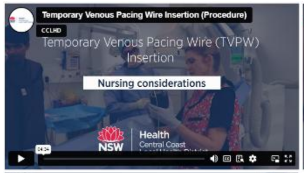<br>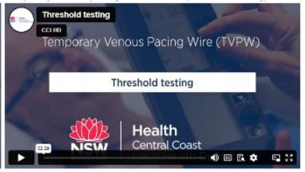<br>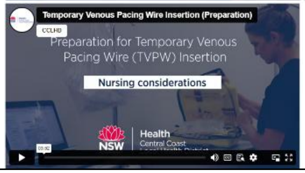
**Pericardiocentesis**	1. Cardiac Tamponade (2.04)<br>2. Procedure (3.23)	• Definition of cardiac tamponade<br>• How it can occur<br>• Definition of pericardiocentesis<br>• Clinical monitoring of a patient undergoing pericardiocentesis<br>• Equipment<br>• Preparation<br>• Assisting the medical officer<br>• Monitoring during and post the procedure	https://youtu.be/lB8qRCnht4w?si=imUw3_uz9ohb7Ptw<br>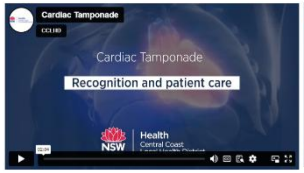<br>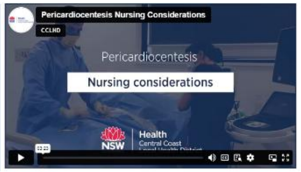
